# Recent progress in the development of upconversion nanomaterials in bioimaging and disease treatment

**DOI:** 10.1186/s12951-020-00713-3

**Published:** 2020-10-29

**Authors:** Gaofeng Liang, Haojie Wang, Hao Shi, Haitao Wang, Mengxi Zhu, Aihua Jing, Jinghua Li, Guangda Li

**Affiliations:** 1 Medical College, Henan University of Science and Technology, Luoyang, 471023 Henan China; 2 School of Medical Technology and Engineering, Henan University of Science and Technology, Luoyang, 471023 China; 3 School of Environmental Science and Engineering, Nankai University, Tianjin,, 300350 China

**Keywords:** Upconversion, PDT, Biomedical applications, Drug delivery, Bioimaging, aPDT

## Abstract

Multifunctional lanthanide-based upconversion nanoparticles (UCNPs), which feature efficiently convert low-energy photons into high-energy photons, have attracted considerable attention in the domain of materials science and biomedical applications. Due to their unique photophysical properties, including light-emitting stability, excellent upconversion luminescence efficiency, low autofluorescence, and high detection sensitivity, and high penetration depth in samples, UCNPs have been widely applied in biomedical applications, such as biosensing, imaging and theranostics. In this review, we briefly introduced the major components of UCNPs and the luminescence mechanism. Then, we compared several common design synthesis strategies and presented their advantages and disadvantages. Several examples of the functionalization of UCNPs were given. Next, we detailed their biological applications in bioimaging and disease treatment, particularly drug delivery and photodynamic therapy, including antibacterial photodynamic therapy. Finally, the future practical applications in materials science and biomedical fields, as well as the remaining challenges to UCNPs application, were described. This review provides useful practical information and insights for the research on and application of UCNPs in the field of cancer.

## Introduction

Traditional surgery and chemotherapy often lead to infection and recurrently [1]. Biotherapeutics including the emerging photodynamic therapy (PDT), which involves precise treatment of tumor cells by in situ generations of singlet oxygen, have proven to be effective disease treatment techniques. These efficient and promising therapies form a new category in the field of disease therapy [2–4]. Before disease treatment was administered, a variety of imaging technologies were employed for cancer diagnosis, including computed tomography (CT) scan, X-ray, ultrasound, magnetic resonance imaging (MRI), positron emission tomography (PET) scan, and fluorescence imaging. The commonly used imaging probes, such as fluorescent dyes and fluorescent proteins, have gained a great deal of appreciation. Nevertheless, they are plagued with deficient detection sensitivity, fast photobleaching, and high toxicity, and have no treatment effect whatsoever. It was difficult to find a dual functional material that can serve as an imaging probe and a drug carrier simultaneously. As an effective drug delivery carrier, it should satisfy the following criteria: (1) effective drug delivery, (2) good biocompatibility, and (3) good stability of in vivo circulation [5–7]. Recently, the rapid development of nanomedicine has opened up a new way to overcome these shortcomings. Nanobiomaterials have become a hot topic in the biomedical-application fields due to their great potential for formulating anticancer drugs. Engineered nanomaterials, exhibiting enhanced permeability and retention (EPR) effect, biocompatibility, and low toxicity, could be used in biomedical applications, including biomedical diagnosing and disease treatment [8, 9].

These nanomaterials typically possess unique optical, magnetic, and acoustic properties, which render them a universal tool in biomedical fields such as bioanalytical science, biomedical imaging and imaging-guided therapy [10–12]. Light-responsive nanomaterials such as quantum dots, metal nanoparticles, have become the optimal candidates because of their advantages including better optical properties i.e. brighter luminescence, small size, good biocompatibility, easy modifications, high sensitivity and higher photostability. These merits enabled the sensitive detection of biological samples and the labeling of reporter molecules [13, 14]. Typically, however, the use of these light-responsive nanomaterials has several limitations, such as background fluorescence interference, easy photolysis, weak penetration, shortly fluorescence time, and long-term toxicity [15, 16]. Upconversion luminescent nanoparticles (UCNPs) exhibit anti-Stokes luminescent, which can convert the near-infrared (NIR) light into visible light. UCNPs have high chemical stability and can be used as fluorescence probes in a variety of complex organisms, facilitating the bioassay process. Moreover, UCNPs could be easily functionalized by linking specific targeting ligands (e.g., peptides, antibodies and small-molecule drugs) to the surface and could be used as probes to detect and target specific cells with high sensitivity and selectivity [17, 18].

Luminescent nanoparticles are effectively used as optical imaging guidance for tumor therapy [19]. UCNPs are an upgraded alternative to traditional optical imaging materials due to several other advantages, such as weak photobleaching, low self-illuminating background fluorescence, deep tissue penetration, and minimal photodamage [20–24]. Moreover, as a new generation of imaging agents, UCNPs are widely used in PDT, upconversion luminescence (UCL), MRI, X-ray CT imaging, photoacoustic (PA) imaging, NIR thermal imaging, up-conversion luminescence imaging, photothermal therapy (PTT), chemotherapy and radiotherapy. Increasing studies have focused on their application in PDT, because they can act as a photosensitizer in photodynamic therapy [25–30]. In addition, they can be applied as drug carriers for bioimaging therapeutic drugs or genes (such as doxorubicin, siRNA, DNA, and microRNA) or photosensitizers. Our group has developed a UCNP-based miRNA delivery vector and achieved excellent gene therapy effect on in vivo and in vitro colorectal cancer models [31]. More importantly, UCNP as the protagonist of antibacterial photodynamic therapy (aPDT) has played a pivotal role in the treatment of bacterial infectious diseases, such as periodontitis and *Staphylococcus aureus* infections, that are intractable and difficult to eradicate [32]. The development of nanomaterials that exhibit both good UCL efficiency and good functionality is undoubtedly a challenge for material scientists, physicists and chemists. Moreover, methods for applying such nanomaterials with unique UCL characteristics to biological, medical, and imaging detection will also attract the interest of biologists and medical scientists in the field of detection.

Although UCNP nanosystems designed for cancer imaging or drug delivery have been summarized in several review articles, a review focused on multifunctional UCNPs designed for both imaging and drug delivery in therapeutics is still lacking. Herein, we systematically summarized the recent progress in the development of UCNPs and introduced the fundamental aspects of UCNP. We then focused on their applications in drug delivery and PDT therapy and the construction of targeted drug delivery and aPDT applications in conjunction with related research. Finally, we highlighted the remaining challenges and speculated on future practical applications of UCNPs in the fields of materials science and biomedical engineering.

## The fundamentals of UCNP composition

**Fig. 1:**
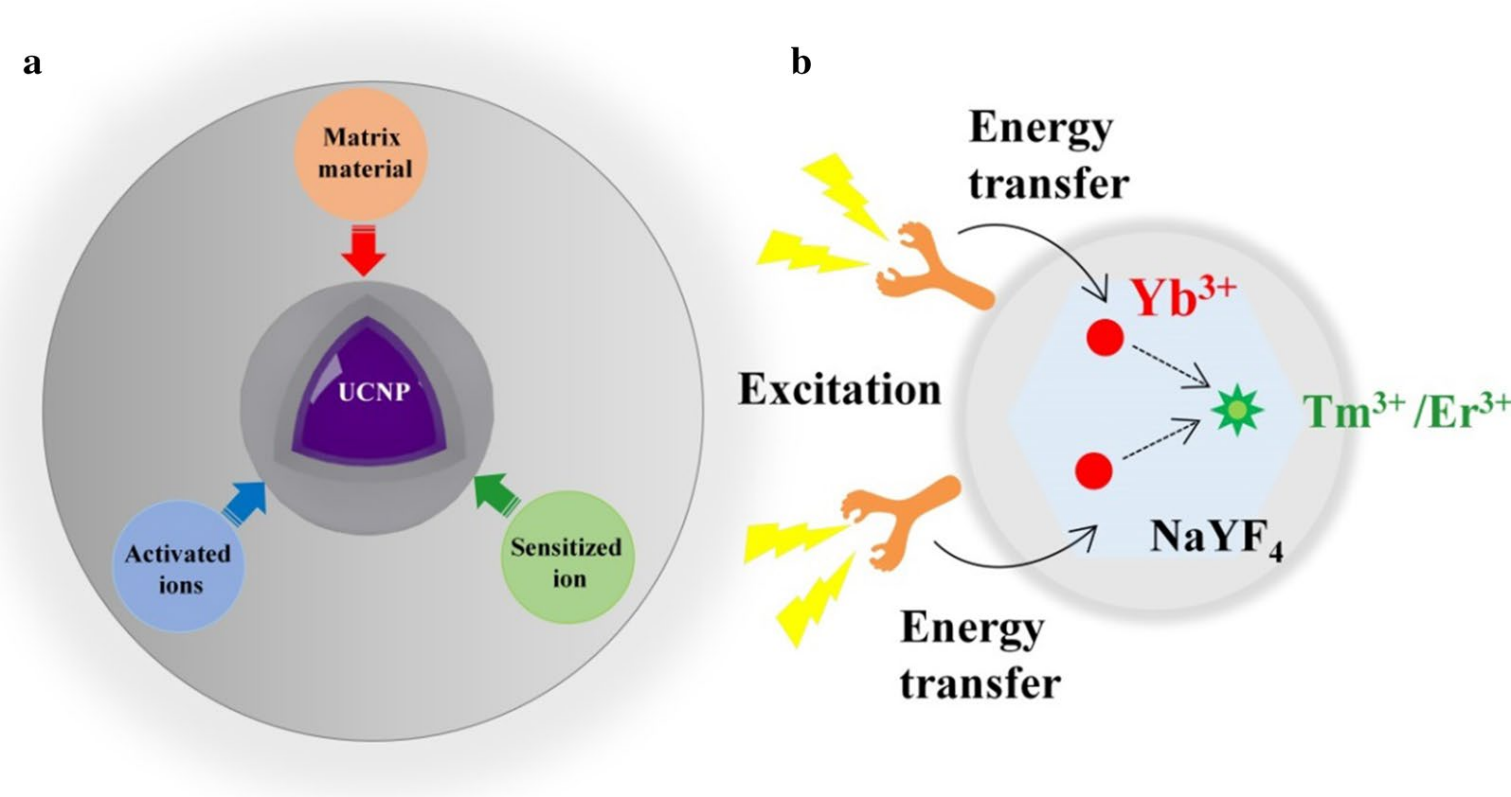
**a** The basic composition of UCNPs and **b** schematic illustration of the mechanism of organic dye-sensitized UCNPs

The unique optical properties of UCNPs are highly dependent on their material composition. Figure 1a illustrates lanthanide-based UCNPs in terms of their matrix material, sensitized ions, and activated ions [33, 34]. The activated ions mainly provide luminescent centers; the sensitized ions absorb NIR light (the absorbed light energy can be transferred to the activated ions to facilitate the emission of light); and the matrix mainly provides a crystalline host lattice structure for activating ions and sensitizing ions to the correct place, giving it the appropriate light conditions. As Fig. 1b shows, attaching an organic dye to the surface of the doped upconverted nanoparticles by electronic interaction and physical absorption can be used to collect the excitation light so that the excitation wavelength can be effectively extended [35]. Moreover, numerous studies have shown that the doping ratio of different ions will also affect the excitation and emission wavelength of UCL (Table 1).

**Table 1:** The doping ratio of different ions of UCNP and their major emission wavelength

Dopant ions and compositional ion				Major emissions (nm)			References
Host lattice	Sensitizer	Activator	Shell	Blue	Green	Red	
β-NaYF_4_	20%Yb	2%Er		450, 476	520	654	Ref. [51]
	20%Yb	0.2%Tm			540		Ref. [38]
	20%Yb	2%Ho			541		Ref. [38]
β-NaYF_4_	25%Yb	0.3%Tm	20%Yb, 2%Er	450	520	653	Ref .[52]
				475	540		
Li^+^ doped β-NaYF_4_	20%Yb	0.5%Tm		452, 479		650	Ref.[39]
Mn^+^ doped β-NaYF_4_	20%Yb	2%Er				657	Ref. [53]
β-NaYF_4_	18.6%Yb	2.2%Er	TRITC-SiO_2_	407			Ref. [40]
	25%Yb	0.3%Tm	SiO_2_	450, 479	521, 539	651	Ref. [54]
	25%Yb	0.3%Tm	FITC-SiO_2_	450, 479	521, 539, 580	651	Ref. [41]
	25%Yb	0.3%Tm	QD-SiO_2_	450, 479	540	605	Ref. [41]
α-NaYF_4_	20%Yb	2%Er		411		660	Ref. [55]
	20%Yb	0.2%Tm		450	540	644	Ref. [42]
	20%Yb	0.2%Er		475	525	693	Ref. [42]

The materials commonly used to synthesize UCNPs mainly include oxides, fluorides and chlorides [36–39]. The choice of metal-fluorides-based host materials has demonstrated dependable chemical stability, and the dopant ions used in the materials were a necessary condition for a stable host lattice [40]. Among many host matrix materials reported, NaXF_4_ is the most common choice for the preparation of high-quality lattice upconversion materials due to its advantages of high chemical stability and low photon energy [41, 42]. The most critical parameter in the consideration of UCL efficiency is the cross-section of the sensitizer ions used to absorb NIR; generally, most lanthanide ions had very small absorption cross-sections in the NIR spectral region, which cannot produce sufficient UC efficiency [43]. However, Yb^3+^ or Nd^3+^, which have larger absorption cross-sections, can completely transfer the absorbed energy to adjacent excited ions in the crystal lattice, which may eliminate the problems associated with the weak absorption of activator ions [44, 45]. The most common activating ions for the lanthanide ion Yb^3+^ in combination with the UC system are Er^3+^, Tm^3+^ or Ho^3+^ and so on. Activated ions are essential to the entire UC emission process [46]. The activator is responsible for emitting visible light and ultraviolet light from NIR light absorbed by sensitized ion conversion, making Er^3+^ or Tm^3+^ are the best candidates because of their long-lived intermediate energy states and because it is convenient to move them from the excitation of their intermediate state to the higher state. When used in combination with Yb^3+^ ions, they produce relatively high UCL efficiencies, as is the case with the currently popular NaYF_4_:Yb, Er/Tm, NaYF_4_:Yb and Er/Tm, used as host matrix, sensitizer and activator, respectively [47].

**Fig. 2:**
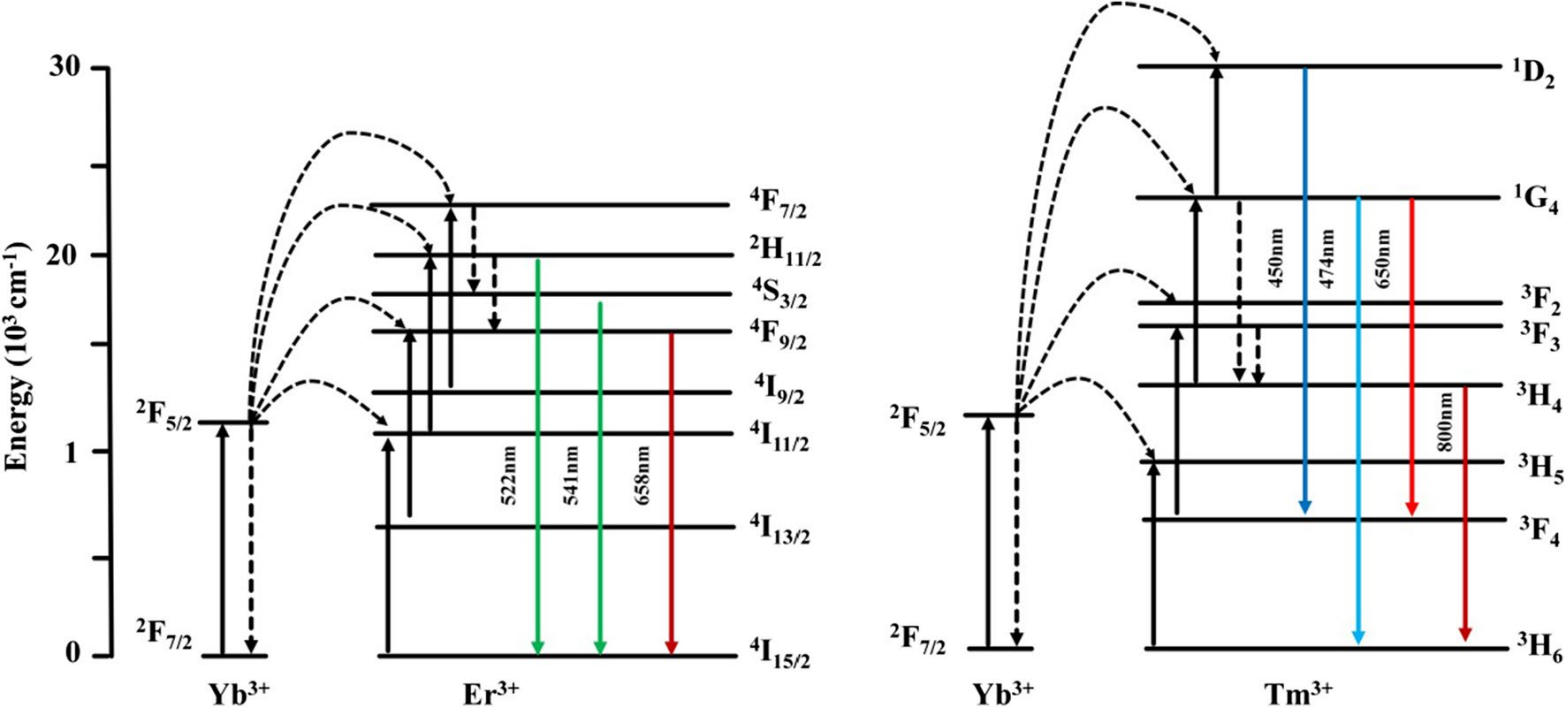
Upconversion energy transfer mechanism based on Yb^3+^ and Er^3+^, and on Yb^3+^ and Tm^3+^ under 980 nm excitation

Sensitized ions (such as Yb^3+^) usually maintain only one excited 4f state. The electronic transition energy in the energy states of individual f–f state resonated with 980 nm radiation [48]. The energy gap in this transition overlaps strongly with many of the f–f electron transitions to the lanthanide activator ions used in the UC system, and then the efficient fluorescence resonance energy transferred will happen [49]. For instance, following the activation of Yb^3+^, an energy transfer from Yb^3+^ to activators (such as Er^3+^ and Tm^3+^) occurs, the energy transfer processes for Yb^3+^–Er^3+^ and Yb^3+^–Tm^3+^ systems are illustrated in Fig. 2. The UC luminescence process of Er^3+^-activated Yb^3+^ can be divided into three parts: first, the conversion of the ground state ^2^F_7/2_ to ^2^F_5/2_ of Yb^3+^, followed by the transfer of the f–f state photon energy to the ^4^S_3/2_^2^H_11/2_, and ^4^F_9/2_ to ground state ^4^I_15/2_, to induce upconversion emissions at 658, 541, 522 nm, respectively. Similarly, the Yb^3+^–Tm^3+^ system emissions at 800, 650, 474 and 450 nm correspond to the radiative transitions from ^3^H_4_–^3^H_6_, ^1^G_4_–^3^F_4_, ^1^G_4_–^3^H_6_, ^1^D_2_–^3^F_4,_ respectively [50].

### Mechanism

**Fig. 3:**
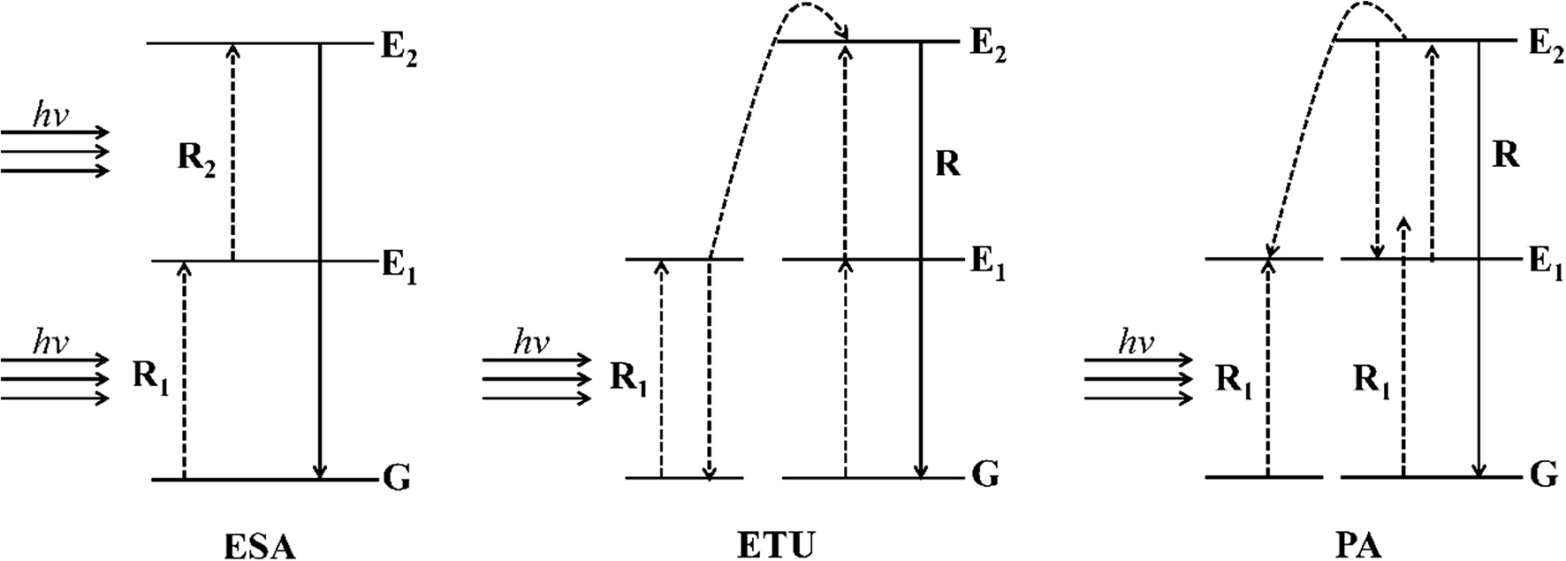
Main upconversion processes of doped UCNP

Abundant energy states of lanthanide-based ions endow the upconversion nanoparticles with enormous chances for the up-energy transfer processes. These complex energy transfer systems can be divided into three processes: the excited-state absorption (ESA), energy transfer upconversion (ETU), and photon avalanche (PA) [56–58]. The fluorescence mechanism of UCNPs based on the principle of the two-photon or multiphoton process of UCL is illustrated in Fig. 3.

Bloem et al. proposed that the excited-state absorption process in ESA is based on the principle that the same ion reaches the higher-energy excited-state energy level from the ground state (G) through continuous multiphoton absorption, which is the most basic UCL process [59, 60]. A photon of the luminescence center of the ground level G absorbs a photon transition to an intermediate metastable level E_1_ which the vibration energy of the photon exactly matches the energy interval of the intermediate metastable level, and the higher excited-state level the ion at the metastable intermediate level jumps to a higher excited-state level E_2_ by absorption of the photon energy to form a two-photon absorption. Moreover, if the energy matching requirement is satisfied, the ion at the excited state level is also likely to undergo higher excited states transition to form three-photon or four-photon absorption [61].

The energy transfer from the ETU process is a form of UCL that occurs when the same or different types of ions interact. The principle of luminescence is that the energy difference between the excited-state and the initial state of energy of the sensitized ion and the activating ion is the same and that when the distance between them is close enough, the electrons in the activating ion jump to a higher excited-state energy level, and resonance can occur between the two and produce energy, which is called energy transfer [62]. There are three main modes of ETU delivery: continuous energy transfer, cross-relaxation and cooperative upconversion.

The PA-based upconversion process is divided into three main steps [63]. First, an electron in ground state ions reaches the E_1_ level of the excited state through nonresonant ground state absorption and then reaches the higher energy level E_2_ through the resonance excited-state absorption process. Finally, the level E_2_ interacts with the neighboring ground state, and the ions undergo cross-relaxation of energy transfer (or ion relaxation process), which results in two ions reaching the intermediate level E_1_. This iteration eventually leads to an exponential increase in the number of electrons at the E_2_ level over time.

## Synthesis and functionalization of upconversion nanomaterial

### Synthesis of UCNP

A plethora of synthetic methods have been developed to prepare lanthanide-doped UCNPs. Because monodispersed nanoparticles smaller than 100 nm usually exhibit good biological performance for biomedical applications, the most commonly used methods for synthesizing UCNP nanoparticles are hydrothermal/solvothermal synthesis, sol–gel methods, and ionic liquid-based methods (Table 2) [64, 65]. Herein, we briefly introduce four representative methods which have been utilized to synthesize UCNPs with uniformly controllable size and high UCL efficiency.

**Table 2:** Main synthetic method of UCNP and advantages and disadvantages

Main synthetic method	Examples and refs	Size range (nm)	Advantages	Disadvantages
Thermal decomposition method	NaNdF_4_ [44] β-NaErF_4_ [68]	50–500	High-quality, uniform size	Intermediate toxicity, high cost
Microemulsion method	LaF_3_ [99] NaYF_4_ [85],	40–500	Easy to operate, narrow size, high stability,	Calcination or annealing usually required
Phase transfer hydrothermal synthesis	(La-Dy)VO_4_ [100], YVO_4_ [100], NaYF_4_ [64],	10–1000	Good dispersion, simple procedures, tunable size	Specialized reaction vessels are needed
Sol–gel processing	GdVO_4_ [101] ^01^	30–600	Cheap raw materials, simple procedures	Broad particle size and unsuitable for bioapplication

#### Thermal decomposition method

The thermal decomposition method is a top-down method that is improved based on the traditional solvothermal method [28, 66, 67]. The main process is to first prepare the organometallic complex, then dissolve it in an organic solvent added with a stable surfactant, and short-term high-temperature thermal decomposition under the protection of the atmosphere, finally prepare the target product. Xie et al. introduced a high-temperature thermal decomposition method to prepare controllable Nd^3+^-doped UCNPs [44]. The results confirmed the feasibility of using Nd^3+^-sensitized core–shell nanoparticles, and the prepared nanoparticles have the same up-conversion characteristics as Yb^3+^-doped UCNPs. UCNP synthesized by the thermal decomposition method can also be applied to multi-functional application platforms. He et al. prepared UCNP with excellent emission wavelength by thermal decomposition method and loaded with photosensitizer for photodynamic therapy of three-dimensional (3D) HeLa cell spheroids [68]. The multifunctional PDT platform has been evaluated to confirm the feasibility of widely biomedical applications. Similarly, Martinez et al. used methanol-assisted thermal decomposition to synthesized highly monodisperse 808 nm excited UCNP, which was tested in vitro PDT treatment [69]. The results explain that UCNP also has excellent colloidal stability and ROS activity in complex biological media. Although the thermal decomposition method can obtain high-quality UCNP with uniform particle size and monodisperse, its preparation precursor has the disadvantages of instability, toxicity, and high preparation cost and soon.

#### Phase transfer hydrothermal synthesis

**Fig. 4:**
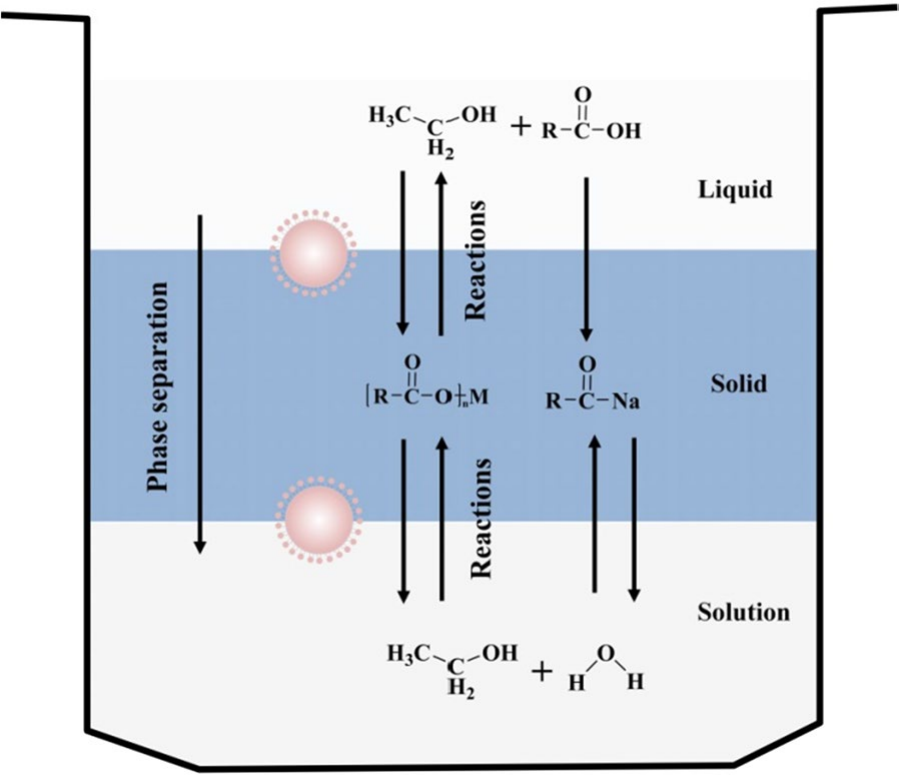
Diagram of the liquid–solid-solution phase transfer hydrothermal synthesis mechanism

**Fig. 5:**
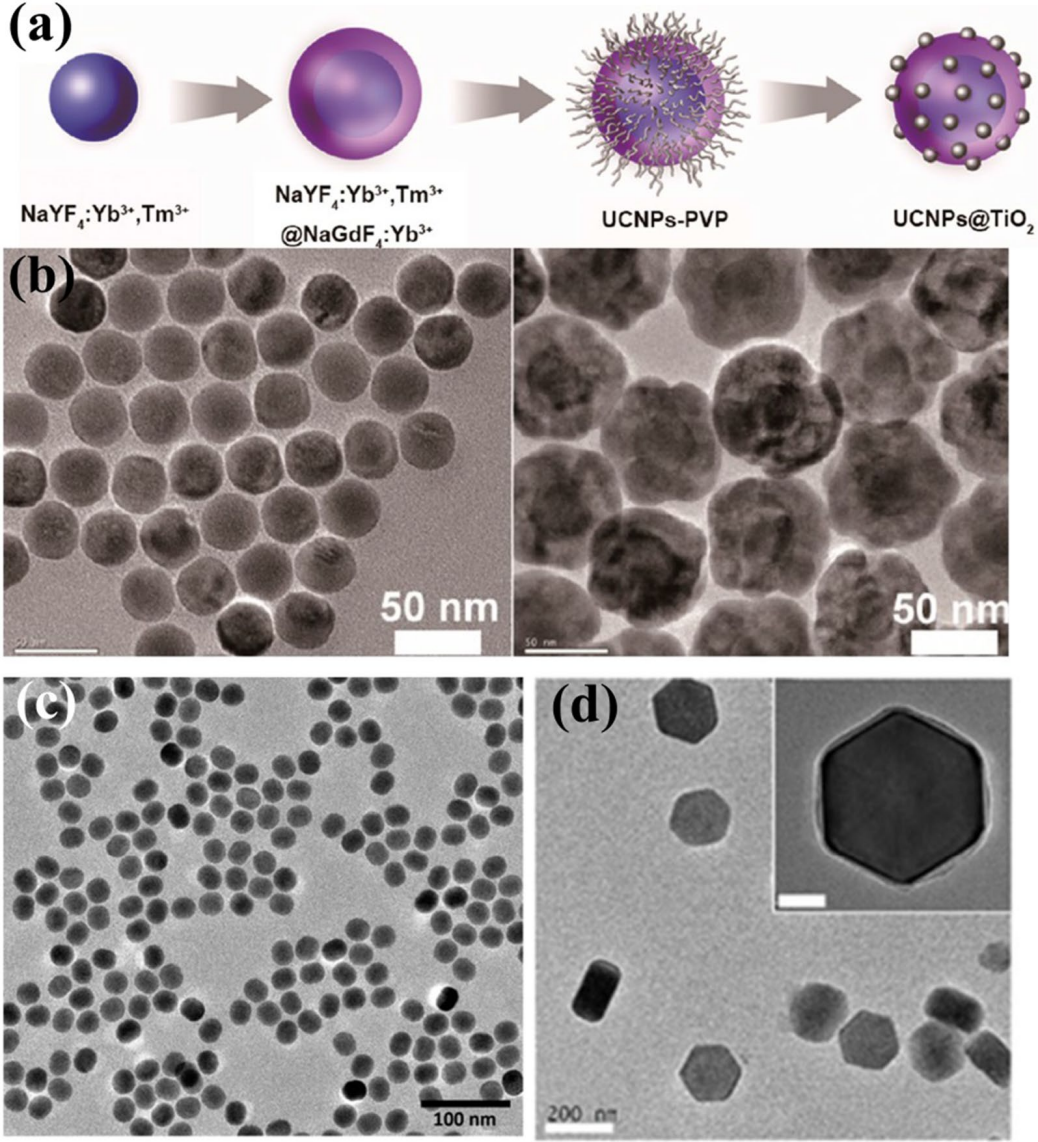
**a** Schematic illustration of the hydrothermal preparation synthetic procedure of UCNPs@TiO_2_ NCs [79]. **b** TEM images of the original NaYF_4_:Yb^3+^,Tm^3+^ cores (scale bar = 50 nm). **c** Sol–gel method for synthesized 2-aminoethyl dihydrogen phosphate-stabilized NaYF_4_:Yb^3+^,Er^3+^ nanoparticles (scale bar: 100 nm) [97]. **d** UPP@ovalbumin were prepared by microemulsion synthesis [98]. (Copyright 2015, 2012 and 2015. American Chemical Society. Reproduced with permission.)

Hydrothermal synthesis provides a cheaper and simpler method for the preparation of UCNPs for biomedical applications [70]. In the preparation stage of hydrothermal synthesis, various types of surfactants and ligands can be added to realize the functionalization of UCNP [71]. Phase transfer hydrothermal synthesis is an emerging synthetic method for the preparation of inorganic materials based on traditional hydrothermal synthesis. The entire reaction process requires high temperature and closed reaction vessel conditions [72, 73], and the solution show a variety of excellent advantages for preparing lanthanide-based UCNPs at room temperature, such as increased of solubility and ion activity [74, 75]. Li et al. provided a detailed description of such carboxylic acids and inorganic metals transfer phase alkylation reaction system based on the thermal brine synthesis mechanism (Fig. 4): first, the lanthanide metal salt-containing heavy metal ions added into the solid phase down to exchange Na^+^ to form an alkyl; the ion exchange process of the chain carboxylic acid complex is then reduced at the liquid–solid or solution-solid phase interface; and the periphery of the nanoparticle is always surrounded by an alkyl chain to form a hydrophobic outer structure [76]. When the nanoparticles grow to a suitable size, they settle down due to the action of gravity, so that the nanoparticles can be collected at the bottom. This method provides more phase selection than traditional preparation processes, and by adjusting the reaction system factors, such as temperature, pH, and processing time, the synthesis of UCNPs can be controlled. Zhang et al. reported the hydrothermal synthesis of NaYF_4_ nanorods, nanotubes, and flower-like nanodisks in a three-phase solution of ethanol, oleic acid and water [77]. By adjusting the pH value of the system, nanosheets, nanowires and the crystal structure of the nanorods were formed; they also found that nanocrystals are more fluorescent in polar solvents than in nonpolar solvents. Li's group used oleic acid to synthesize UCNP in aqueous solution, and then added cyclodextrin into the system to make the nanoparticles hydrophilic and soluble, which enables them to carry hydrophobic drugs and dyes [78]. Zhang et al. designed a novel NIR photoactivated photosensitizer based on TiO_2_ coated UCNP core/shell nanocomposites (UCNPs@TiO_2_ NCs) by using water phase transfer to synthesize UCNPs (Fig. 5). NaYF_4_: Yb^3+^, Tm^3+^ @ NaGdF_4_: Yb^3+^ core/shell UCNPs can effectively convert NIR light into UV emission, which is matched with the absorption of the TiO_2_ shell [79]. However, this method of producing nanoparticles is inefficient and time-consuming, and the synthesis process is difficult to control and costly. A great deal of effort has been put into new approaches to synthesizing UCNPs to make the process faster, cheaper, and more efficient, not only for fundamental research, but also for high-tech applications.

#### Microemulsion method

Microemulsion template synthesis refers to the surfactants and raw material involved in the template synthesis method, which uses several aggregated forms obtained after aggregation, such as micelles, vesicles, liquid crystals, and microemulsions, which form a microreactor with a size in the nanometer range [80–82]. The size and internal state of the droplet reactor determine the basic properties of the nanoparticle, and due to the morphological controllability of surfactant soft aggregates, the controllable nanoparticles can be better achieved [83, 84]. A successful example has been reported: UCNP wrapped in ethylenimine polymer synthesized by this method can be used as advanced fluorescent probes to detect Pb^2+^ through the fluorescence quenching process generated by electrostatic interaction with the polymer [85]. Yang et al. combined synthetic UCNPs with carbon nanotubes using surfactants, and further therapeutic effects are evident in genomic DNA damage and the cell cycle [86]. Therefore, the prepared nanoparticles interface had high stability and good dispersion compared with other methods, the addition of surfactant molecules can not only control the size, but also modify the surface of the nanoparticles [87, 88]. However, the microemulsion method needs to remove the surfactant after preparing the nanoparticles. Compared with other methods, the yield of this method is reduced by 20–40% [40], and the nanoparticles have weakened monodispersity, so this method is suitable only for laboratory research [89].

#### Sol–gel method

The sol–gel method generally uses a metal–organic or inorganic solution as the base solution in low temperature solution to prepared inorganic materials or composite materials [90–92]. This method has the unique advantages of uniform mixing of reactants, easy doping of trace elements and low reaction temperature. On this basis, a large number of studies on temperature characteristics of UCNPs have been performed. Peng et al. [93] used the sol–gel method to prepare lanthanide co-doped Na_0.5_Gd_0.5_MoO_4_ phosphors and evaluated them as optical temperature sensors and optical heaters for potential applications. Tang et al. used this method to synthesize La, Gd, and Lu nanocrystals doped with Yb^3+^ and Er^3+^, and compared the temperature-dependent UC behavior and optical temperature sensing characteristics of the fluorescence intensity ratio of these nanocrystals [94, 95]. Prasad et al. prepared a ZrO_2_: Er UCNP based on ZrO_2_, TiO_2_: Er, BaTiO_3_: Er, Lu_3_Ga_5_O_12_: Er and YVO_4_: Yb/Er using an improved sol–gel method [96]. However, it is difficult to achieve the desired UCNP particle size and dispersion level using this method, which impacts the surface modification of the nanomaterial and limits its application in biomedicine.

### Functional modification

**Fig. 6:**
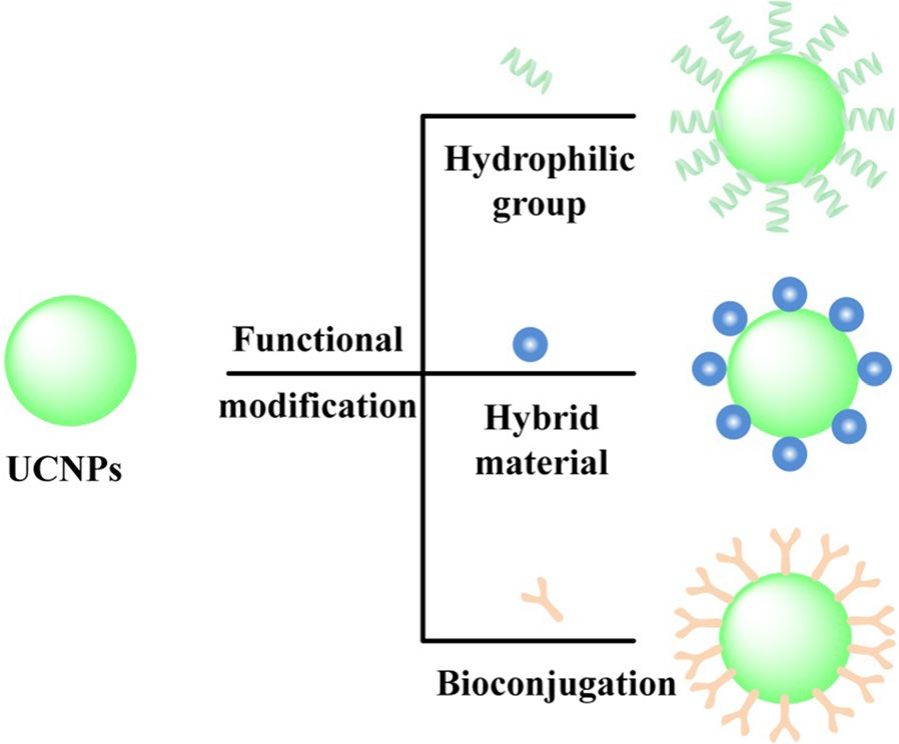
Commonly used methods of functionalizing UCNPs

Usually, subsequent surface modification of nanoparticles is necessary to yield a surface composition suitable for biomedical applications [102]. For example, by changing UCNPs surface ligands to load some hydrophobic drugs, surface charge to adsorb small molecules, and even in combination with some biomolecules for bioimaging and therapeutic applications [103]. Nevertheless, different needs create different modifications, and careful design and optimization of these aspects are essential before UCNPs can be used for bioanalytical applications. A variety of surface modification studies have been reported; we divide the functional modifications into three categories (in Fig. 6): hydrophilic modification, bioconjugation, and hybrid materials [104].

#### Hydrophilic modification

**Fig. 7:**
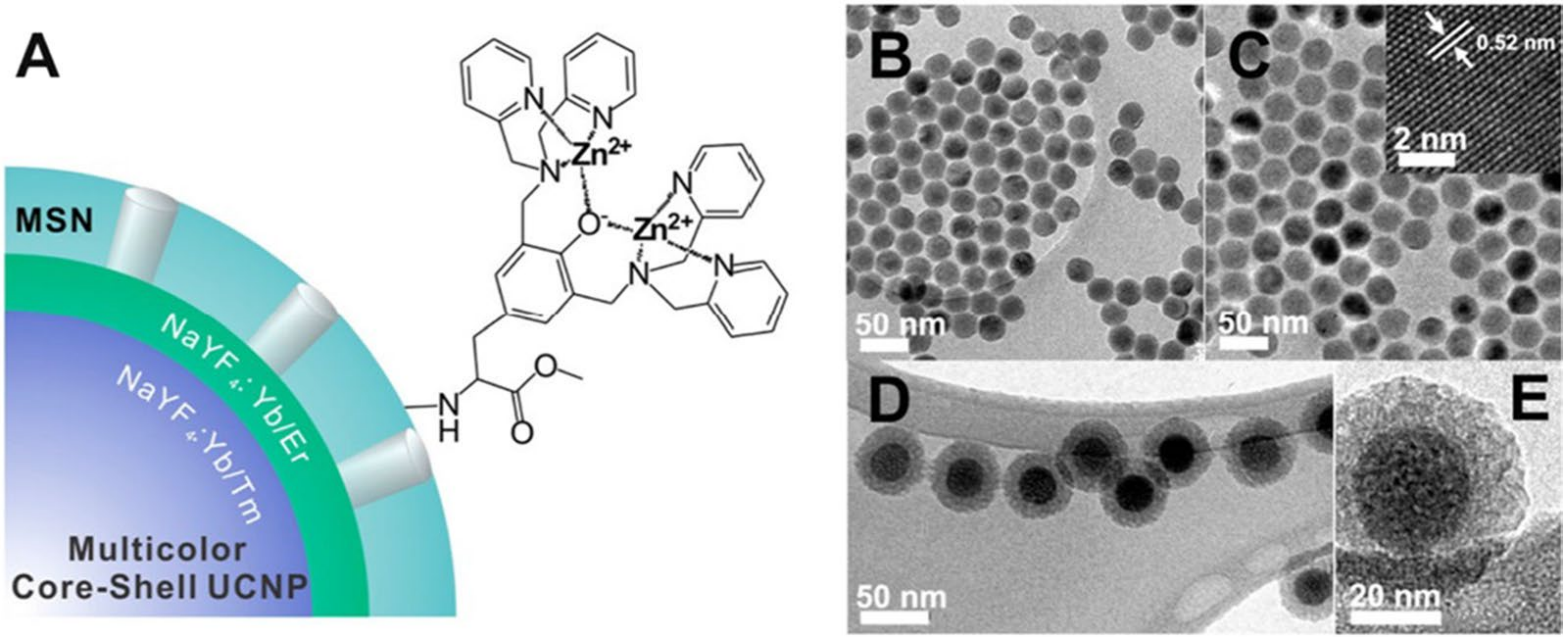
**a** Hydrophilic modification structural illustration of a multicolor core/shell UCNP, **b**–**e** TEM images of the UCNP before the growth of NaYF_4_:Yb^3+^/Er^3+^ shell core and after functionalization of NaYF_4_:Yb^3+^/Er^3+^ with zinc-dipicolylamine analog (TDPA-Zn^2+^) and MSNs [114]. ( Copyright 2015. American Chemical Society. Reproduced with permission.)

The nanoparticle dispersion in an aqueous medium must be stable under long-term preservation to avoid aggregation and precipitation during storage or application [105]. The initially synthesized UCNPs are easily covered by hydrophobic ligands, which directly lead to the inability of UCNPs to form stable water-dispersion systems [106]. In fact, organic solvents have higher phonon energy and energy transitions than water. However, the transfer to an organic solvent leads to fluorescence quenching, and surface hydrophilic modification of UCNPs seems imperative [107]. Methods for producing hydrophilic modifications usually involve adding (1) acidic ligands [108] (2) polymers [109] or (3) chelating agents [110], and these ligands usually have hydrophilic functional groups, such as a hydroxyl group, primary or secondary amine, and carboxylic acid, etc. When citrate acid is used as a growth control agent in hydrothermal synthesis, the resulting UCNP’s shape and size can be controlled and evenly dispersed in aqueous solution by adjusting the ratio [111]. Ethylenediaminetetraacetic acid, which has a high chelating constant for all lanthanide-based ions, and coordinated surface by chelation to stabilize the lanthanide ions in the solution [112, 113]. Because of the presence of primary, secondary and tertiary amino groups of polymers, polyethyleneimine (PEI) can easily coordinate with the lanthanide ions, thereby transforming the growth of the nanoparticles and obtaining pro-aqueous UCNPs [113]. Lai et al. described a hydrophilic functionalization of a zinc-diamine analog (TDPA-Zn^2+^) on the surface of UCNPs (Fig. 7) and the loading of small-molecule drugs or chemotherapeutic agents in the internal mesopores [114].

#### Bioconjugation

**Fig. 8:**
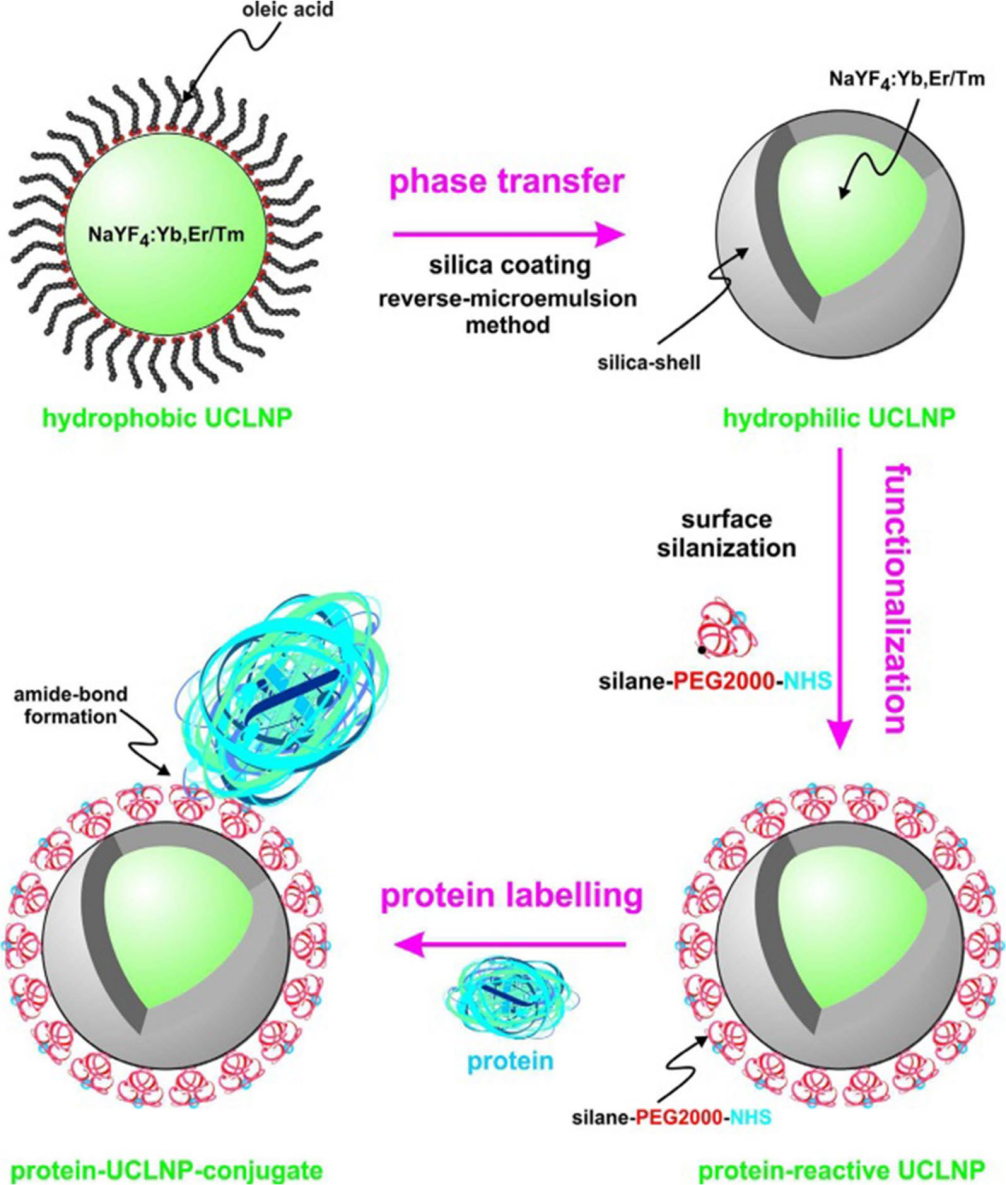
Surface engineering of a UCLNP towards protein-reactive, multicolor upconverting labels

Nanomaterials can be used in bioanalytical applications by modifying specifically identified biomolecules. Table 3 presents some biomolecules which can be used as specific recognition elements of UCNPs through preferential surface modification. Many biomolecules, such as streptavidin, are negatively charged proteins that can easily coordinate with UCNP surfaces but cause nanoparticle aggregation. Kamimura et al. reduced the nonspecific binding by co-simulating PEG-b-PAA and streptavidin on the surface of the UCNP, and usually, in the four binding sites of streptavidin, at least one of them could be used for biotin binding [115]. Recent research has found that the surface-modified ligand on UCNPs can be converted to a new functional group to affect the subsequent bioconjugation step. Ligands commonly used for attachment are maleimides [98], thiols [116], carboxylic acids [117], aldehydes, and amine groups. Figure 8 showed a typical bioconjugation process, which uses a typical amide reaction to achieve the function of engineering a specific protein on the surface of UCLNP [118]. On the other hand, the amine group of the UCNP can be converted into a carboxyl group by a ring-opening reaction of succinic anhydride or glutaric anhydride, and the exposed carboxyl group can then be activated by forming an N-hydroxysuccinimide ester to realize the purpose of the combination [119, 120].

**Table 3:** UCNP modified by biomolecules in biomedical applications

Biomolecule	Examples and refs	Application
Antibody	NaYF_4_:Yb,Er [130]	LRET system
	NaYF_4_:Yb,Er [131]	Photodynamic therapy
DNA/RNA/MiRNA	NaYbF_4_:Yb,Er [109]	Delivery and transfection of siRNA
	NaYF_4_:Yb,Tm [108]	Specific binding site
Protein/peptide	NaYF_4_:Yb,Er [132]	In vivo imaging
	NaYF_4_:Yb,Tm [110]	Proof of principle
Folic acid	NaYF_4_:Yb,Er [133]	Tumor targeting/imaging
Avidin	NaYF_4_:Yb,Er/ NaYF_4_:Yb,Tm [134]	Assay application

#### Hybrid materials

**Fig. 9:**
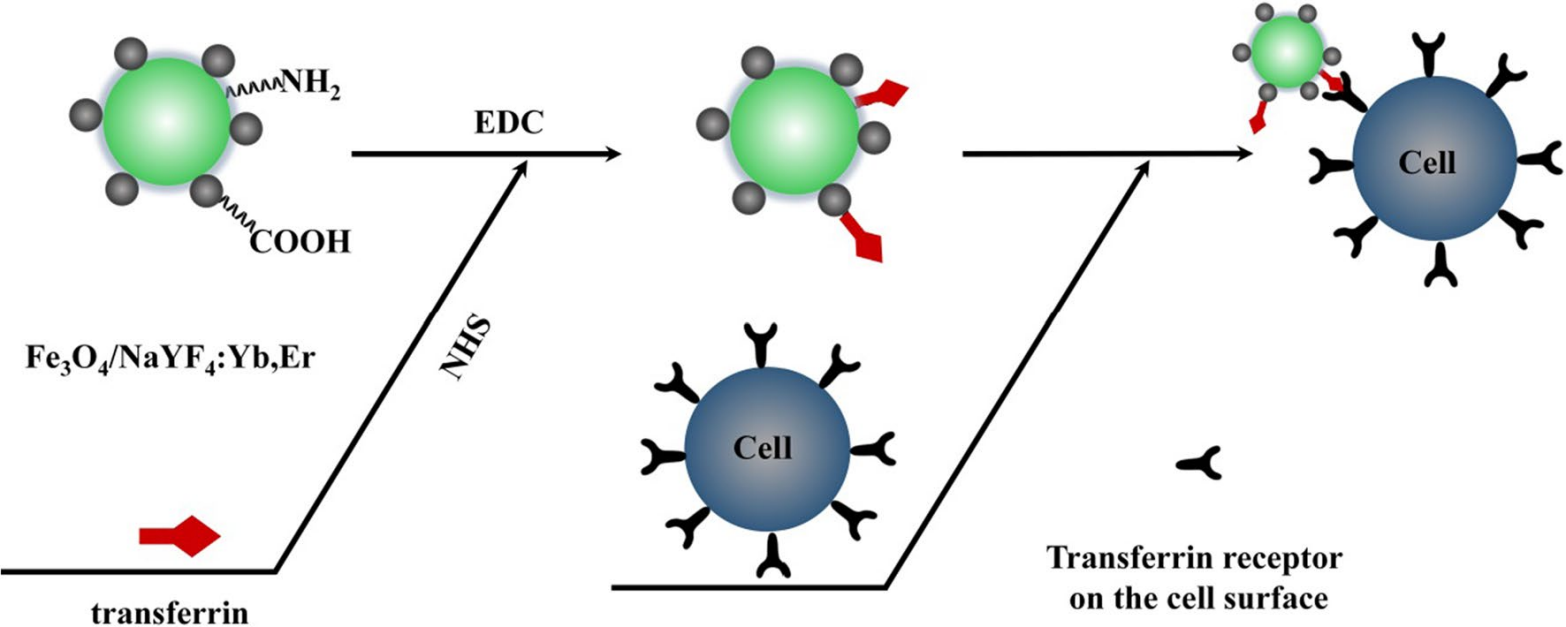
Schematic diagram of the chemical coupling method to combine Fe_3_O_4_ with NaYF_4_ nanomaterials for biomarker HeLa cells [120]. ( Copyright 2010. The National Center for Biotechnology Information. Reproduced with permission.)

The combination of UCNPs with other nanomaterials can formation hybrid materials for the multifunctional applications. In some specific circumstances, the hybrid materials can not only enhance UCL, but also own the characteristics of materials. UCNPs can also be doped with paramagnetic agents such as Gd^3+^, especially in combination with superparamagnetic Fe_3_O_4_ nanoparticles, which can be applied to multifunctional biomedical platforms. As shown in Fig. 9, the synthetic process of magnetic UCNPs was prepared by EDC/NHS activation and the binding of UCNP surface affinity to Fe_3_O_4_ nanoparticles [120]. The hybrid nanomaterials make a difference in the separation and purification of biomolecules or the design of multimodal bioimaging probes. Noble metals such as a silver shell modified on the surface of UCNPs can enhance UCL efficiency by surface plasmon coupled emission to produce a multimodal hybrid material, which can be used for photothermal therapy [121]. By adjusting the thickness of the silver layer, the wavelength of the UCNP surface plasmon resonance can be adjusted to 980 nm, and bioimaging by upconversion emission can be performed simultaneously under single wavelength illumination [122]. Similarly to the magnetite nanoparticle modification method, silver nanoparticles occupy the thiol group on the surface of the UCNP for nucleation behavior.

## Upconversion nanomaterials for biomedical applications

**Fig. 10:**
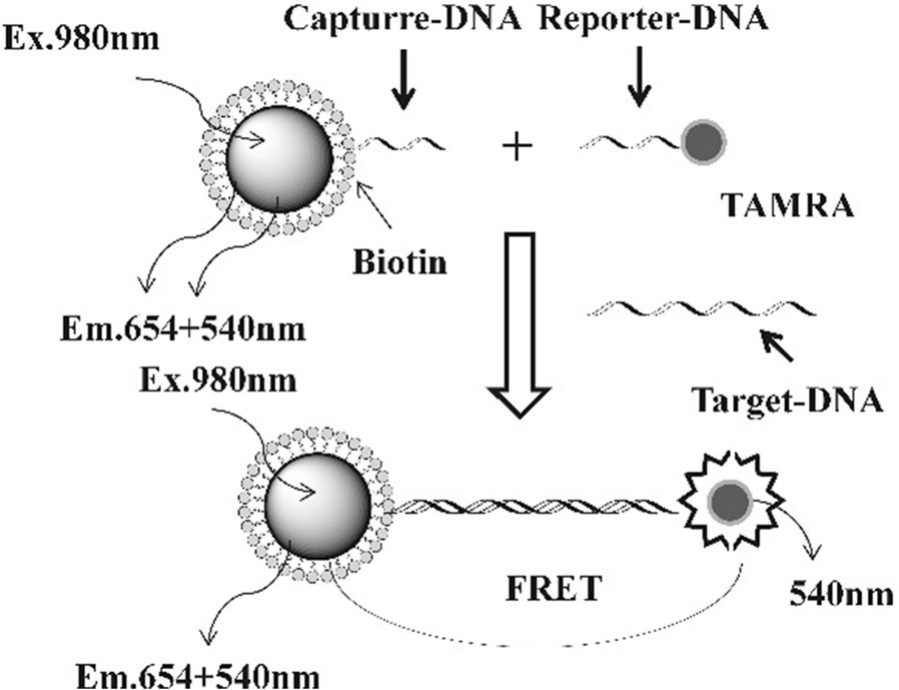
Schematic illustration of the design principle of DNA nanosensors based on upconversion fluorescent resonance energy transfer

The morphology and size of nanoparticles (NPs) result in (1) prolongation of the blood’s circulation time and (2) low endotoxin, which can be absorbed and cleared in the cells by endocytosis. Thus, NPs with a small molecular size can be used as a powerful tool for biomedical research and clinical diagnosis and treatment. UCNPs have been adopted in a wide range of biomedical applications, due to their unique NIR excitation, high luminescence stability, and high detection sensitivity, especially in biosensor, bioimaging, and disease treatment. In biosensing applications, UCNPs can be used in the detection of infectious bacteria, pathogens, and viruses in food residues, especially UCNPs have been extensively studied in immunochromatographic assays, which is one of the best devices for instant diagnosis [123–125]. Figure 10 illustrates the use of UCNPs as energy donors and the subsequent selection of appropriate energy receptors to achieve the detection of targets through luminescence resonance energy transfer (LRET), which has been applied extensively in early disease diagnosis [126]. Li’s group initiated the research on the FRET phenomenon, which is based on UCNPs designed for biological detection [127]. The biotin-modified UCNPs and gold nanoparticles form an energy-donor–acceptor system, when UCNPs are attached to gold NPs; the UCNPs will undergo a quenching effect. Subsequently, with the increased number of Au NPs on the UCNP surface, the quenching may become increasingly intense, so that the concentration of avidin can be detected with a limit of 0.5 nM. In addition to the above biosensing applications, lanthanide-based UCNPs have also been used as imaging agents and therapeutic carriers, with great potential for biomedical applications [128, 129].

### Biological imaging

Bioimaging probes are usually composed of organic fluorophores or fluorescent proteins, but they produce strong background fluorescence in the visible light region and have poor light stability, which greatly limits their biomedical applications [135–137]. Quantum dots (QDs) have high photostability, it is difficult to make extensive use of them in vivo due to their toxicity and side effects. In contrast, lanthanide-based UCNPs have unique advantages, such as stable chemical properties, high-intensity light emission efficiency, deep penetration, and no background fluorescence, which make them an ideal biomarker for transcending organic fluorescence and QDs [138]. UCNPs can emit visible light under the excitation of NIR, which can avoid the tissue damage caused by the excitation light, and the application of the NIR excitation light increases the photon’s penetration limit in tissues.

#### Upconversion luminescence imaging

**Fig. 11:**
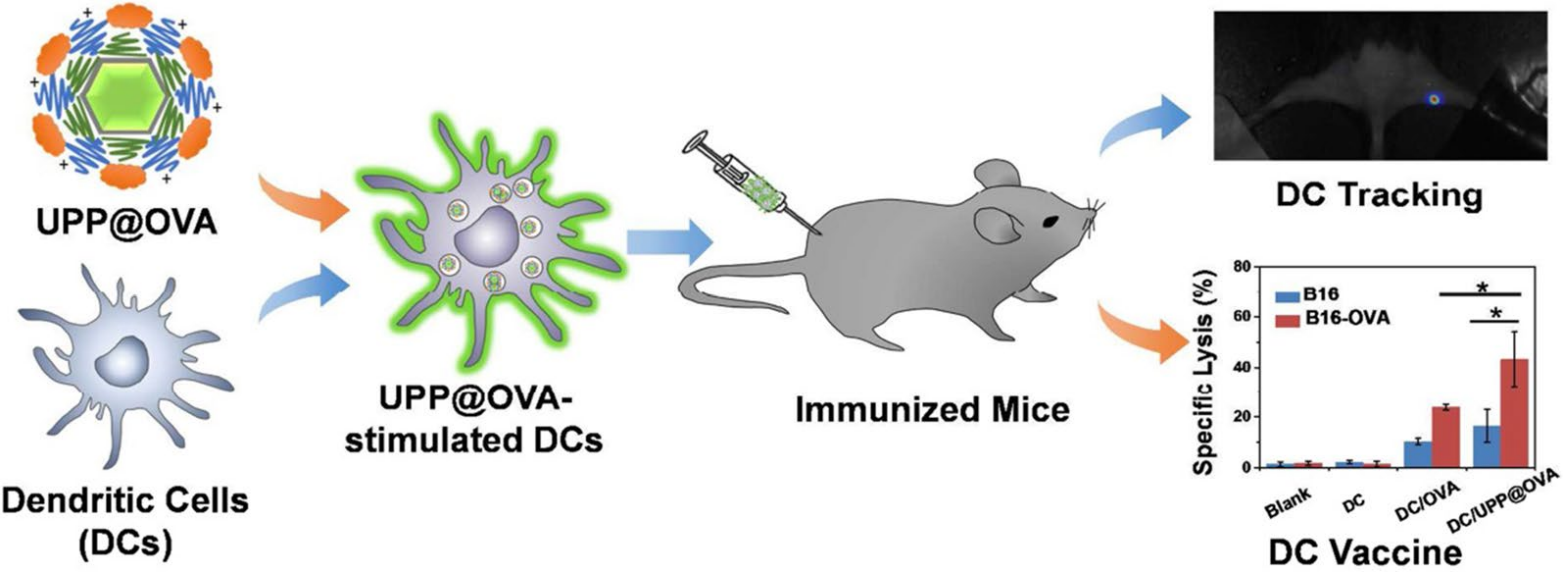
Schematic illustration of antigen-loaded UCNPs for dendritic cell (DC) stimulation, tracking and vaccination in DC-based immunotherapy [98]. ( Copyright 2015. American Chemical Society. Reproduced with permission.)

UCL bioimaging is based primarily on visible (red, green or blue) emissions from Er/Ho/Tm doped UCNP, and the emission spectrum ranges cover from blue to green fluorescence [139]. In the spectral range of 400–700 nm, cells have a strong absorption band and are therefore often used to monitor single molecules and growth processes in living cells. The work of Chatterjee et al. included the use of PEI-coated NaYF_4_:Yb, Er for cancer cell imaging. Moreover, strong green UCL could be observed on the cell membrane by fluorescence observation under NIR excitation [140]. Early NIR to visible UCL bioimaging was difficult to achieve the visible millimeters of tissue penetration depth. However, Zhao and colleagues demonstrated UCL imaging with considerable tissue depth using NaYF_4_:Yb, Er NPs as a luminescent probe for nude mice achieving a penetration depth of 1 cm for the first time [141]. In addition, Liu and colleagues compared UCL imaging of pork muscle tissues at different depths (0–1 cm) by injection of polymer-modified NaYF_4_:Yb, Er and KMnF_3_:Yb, Er. For NaYF_4_:Yb, Er the image can be detected at a depth of about 0.5 cm, while KMnF_3_ has a very strong red emission which can sense a tissue depth of 1 cm [107]. In Xu et al.'s work (Fig. 11), antigen-loaded UCNPs were used to label and stimulate dendritic cells (DCs), and the UCNP-labeled DCs achieved high-sensitivity in vivo UCL imaging; finally, a strong antigen-specific immune response was induced by the successful postinjection of DC vaccines [98].

#### Tumor targeting imaging

**Fig. 12:**
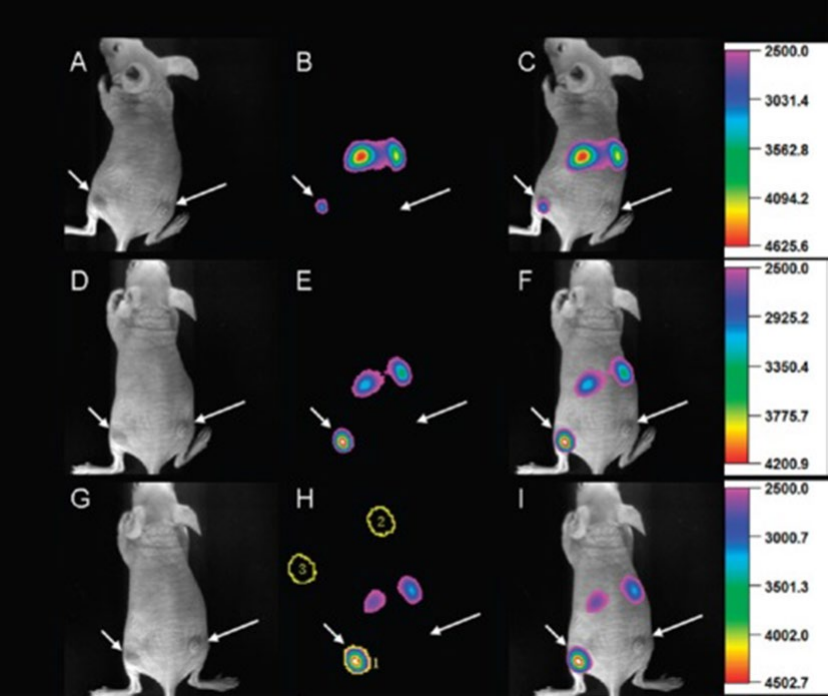
Time-dependent in vivo upconversion luminescence imaging of subcutaneous U87MG tumor (left hind leg, indicated by short arrows) and MCF-7 tumor (right hind leg, indicated by long arrows) borne by athymic nude mice after intravenous injection of UCNP-RGD over a 24 h period [146]. ( Copyright 2009. American Chemical Society. Reproduced with permission.)

Tumor targeting is a prerequisite for improving imaging and therapeutic efficacy; the targeting markers specialized for diseased areas are particularly excellent for cancer treatment that selectively kills cancer cells [109, 142, 143]. Emerging targeting technology for the attachment of biomolecules to imaging agents provided high efficiency and low toxicity for molecular targeting. Due to the unique optical properties of UCNP, they are widely used in actively targeted imaging of tumors. Li and coworkers engineered a targeted imaging system based on RGD peptides and αvβ3 integrin receptors (Fig. 12) [144]. They linked RGD peptides to the surface of UCNP to form UCNP-PEG-RGD and found that the nanoparticles could efficiently target U87MG tumors. A strong UCL signal was witnessed in the left hind leg excited by the excitation of 980 nm; however, no noteworthy UCL signal was seen in the other legs. Achieving in vivo specific tumor imaging based on RGD peptide-protein recognition showed that UCNP can be used as molecularly targeted probes for the diagnosis of tumor tissue. Chen et al. [145] applied PEI-modified UCNP to cancer cell bioimaging; the synthesized NaYF_4_:Yb, Er@SiO_2_ nanocrystals were directly applied to MCF-7 cancer cells under 980 nm excitation, and the green luminescence in cells was performed by confocal microscopy.

#### Multi-modality imaging

**Fig. 13:**
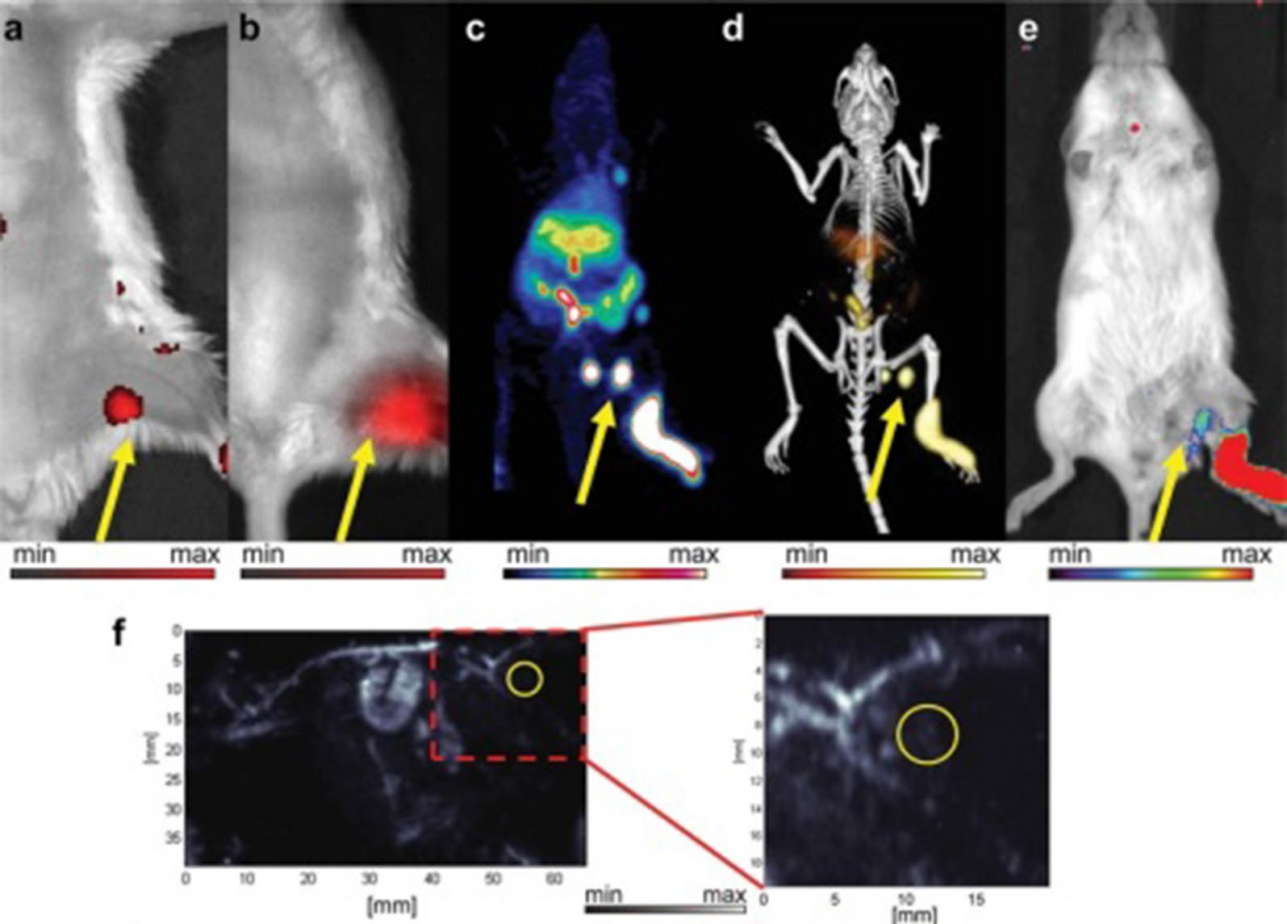
In vivo lymphatic imaging using PoP-UCNPs in mice. **a** Traditional FL and **b** UC images **c** full anatomy PET, **d** merged PET/CT and **e** CL images and PA images **f ** [153]. (Copyright 2015. Advanced Materials. Reproduced with permission)

To obtain accurate imaging information for clinical diagnosis and further practical processing, researchers have expanded imaging applications by introducing other functional components to form nanocomposites, in particular by combining the benefits of two or more nanoparticle imaging techniques to form multimodal biological imaging [147]. UCNP are excellent optical imaging probes and can be used as multimodal contrast agents for multimodal bioimaging, such as UCL/MRI, UCL/MRI/CT, UCL/MRI/PET, UCL/CT and MRI/CT. Here, we highlight the imaging applications of UCL/MRI. MRI is a medical diagnostic technology based on nuclear magnetic resonance (NMR); MRI and UCL imaging are two modes of coordination. When one system is combined with another, the advantages of high spatial resolution and sensitivity are combined to enhance the signal strength of bioimaging. NPs can be combined with UCNP to design bimodal UCL/MRI contrast agents, such as Gd-centered hosts and NaGdF_4_ [148], Gd_2_O_3_[149], GdF_3_[150] and BaGdF_5_[151]. Li and colleagues confirmed that NaGdF_4_:Yb, Er, Tm-PAA NPs exhibited a high relaxation rate (5.6 s^−1^ mM^−1^) and it was clear that the signal enhancement of the UCNP in different organs after comparison [152]. James et al. studied in vivo lymphoid imaging of mice using PEG and chelated radioactive ^64^Cu PoP lipid coated UCNP ( Fig. 13) which was found to exert an active influence in six different imaging modalities. More specifically, PET and CT provided the deepest tissue imaging capabilities, and CL and UC imaging could detect deeper signals for the previous FL [153]. In summary, UCNPs used for manufacturing simple but higher order multimodal imaging agents are feasible and can be used in the development of super-integrated imaging systems [21, 154].

### Therapeutics

Scholars have explored how to shorten the gap between biomaterials and clinical treatment diagnosis. Therefore, the construction of a multifunctional nanomedicine platform for disease treatment has attracted their attention. To meet the needs of both disease diagnosis and in vivo treatment, varieties of UCNP-based nanocomposites have been used as drug delivery systems as well as drug monitoring devices.

#### Drug delivery and release

**Fig. 14:**
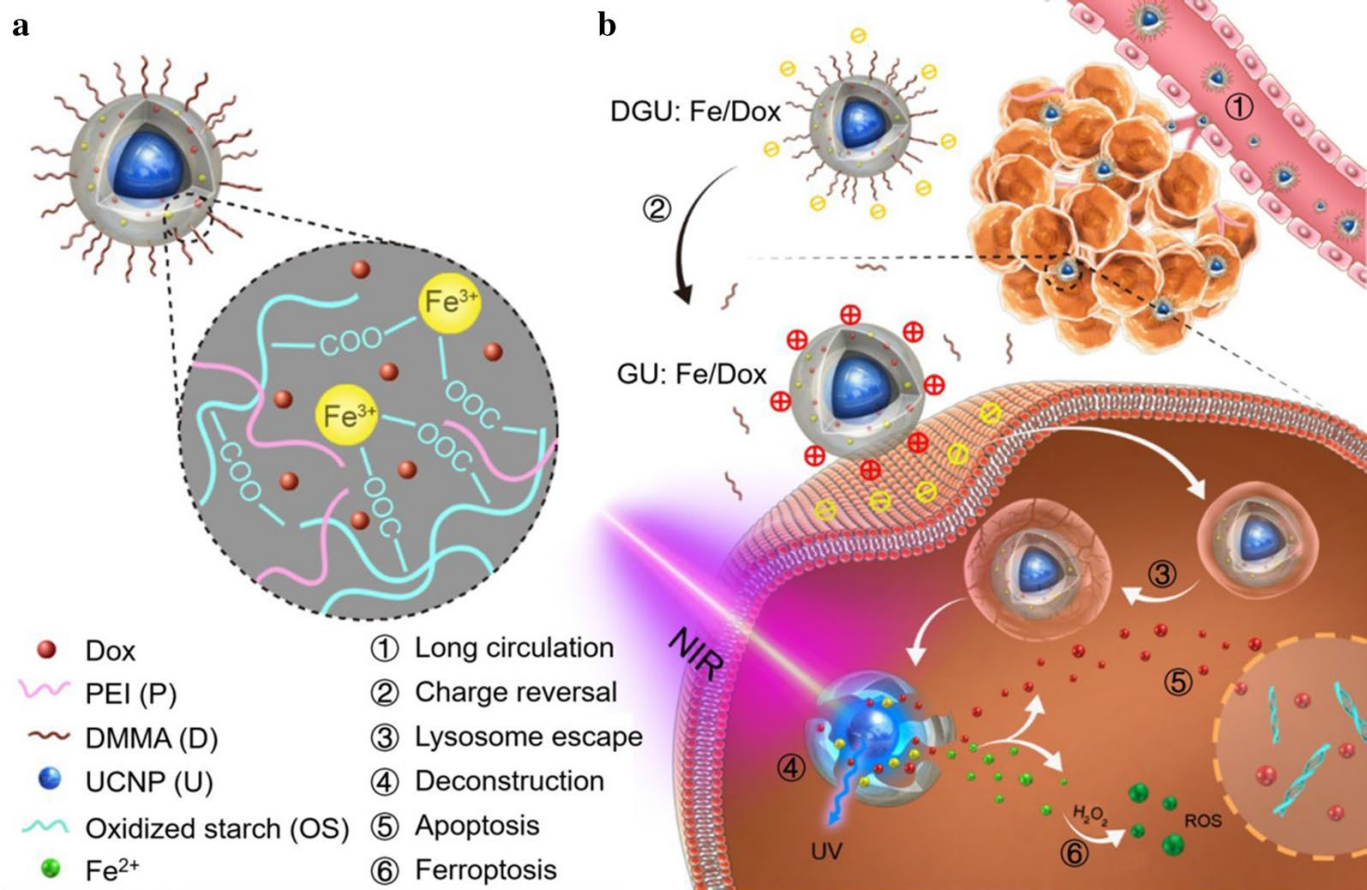
UCNP and DOX were loaded into gel nanoparticles and modified with PEI and DMMA to construct a nanolongan schematic with multiple transformations and corresponding anticancer mechanisms. ( Copyright 2019. American Chemical Society. Reproduced with permission.)

Compared with traditional medicine, UCNP-based drug delivery has obvious advantages such as small particle size, which facilitates the endocytosis of cells to obtain a good therapeutic effect [155–157]. The huge surface area of UCNP can prolong the retention of topical drugs and increase the utilization of drug targeting in the tissues [158, 159]. Even better, UCNP-based composites used as drug delivery systems will enable tracking and efficiency evaluation of drug release in real-time [160, 161]. Researchers have prepared UCNP drug delivery for drug loading, and targeted modification of the delivery surface to release drug molecules and transport them into tumor cells [162]. Duan et al. grafted the target molecule folic acid (FA) onto the surface of PEI-modified UCNP and loaded with camptothecin (CPT)/doxorubicin (DOX) for the targeted cancer treatment at the first time [125]. Liu et al. constructed a multifunctional nanocarrier based on an oleic acid-blocked UCNP, which was loaded with DOX drugs by directly coating NIR-absorbed polydopamine (PDA) shells on the surface of UCNPs and provided five kinds of biological imaging. The function realized the synergistic inhibition effect of PTT and the chemotherapy effect on mouse colorectal SW620 tumors [163]. Lanthanide-based UCNPs can also be induced by NIR light to achieve drug and gene release therapy [164, 165]. Liu et al. constructed a nano-transport system that encapsulated UCNP and DOX in oxidized starch-based gel nanoparticles (Fig. 14); further, PEI and 2,3-dimethyl maleic anhydride (DMMA) were used to decorate them. The results show that multiple modifications can prolong the circulation time of the drug in the body and that the EPR effect provides more opportunities for the drug to reach the tumor site and achieve rapid release of the drug pair [166].

UCNPs used as drug delivery devices can achieve the conventional release process. Lin's group designed a controlled drug release system based on UCNPs that could accelerate drug release by changing pH or temperature conditions [65]. It is worth mentioning that the new light-controlled drug delivery process based on the structure of UCNPs has become a new research hotspot, in particular through the use of NIR-light-induced photo-controlled drug release [161, 167]. NIR-UV-based UCNPs were used for gene release: after 980 nm excitation, UCNP could release DNA and small interfering RNAs (siRNA) from UV light. The induced photo-controlled drug release inhibited the gene expression of tumor cells to achieve therapeutic effects [138].

#### Gene delivery

**Fig. 15:**
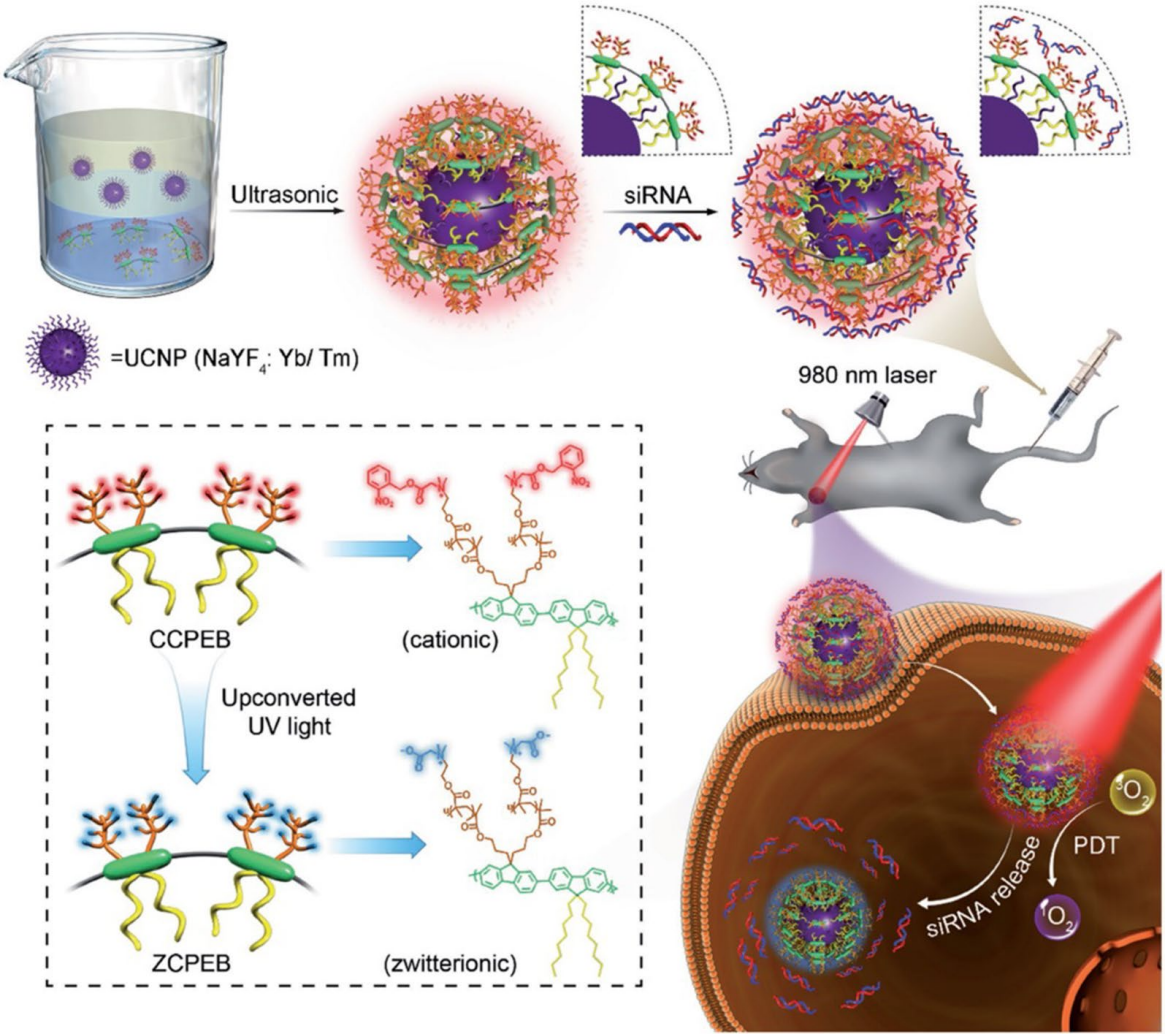
Synthesis route of photo-induced charge-variable cationic conjugated polyelectrolyte brush and its photolytic process. ( Copyright 2017. John Wiley and Sons. Reproduced with permission.)

The key challenge of cancer gene therapy is achieving a precise strike of the therapeutic agents into the solid tumor site. One effective method for accomplishing this goal is to develop efficient gene delivery systems to improve the therapeutic index of drug molecules and minimize the toxic side effects on healthy cells and tissues. UCNPs have been reported to provide comprehensive transport of genes such as DNA and siRNA. The tricky problem of RNAi therapy is how to effectively protect the siRNA biomolecules from the complex physiological environment and how to increase the delivered expression effect. Yang's research team mounted siRNA on the surface of UCNPs in order to attach anti-Her2 antibodies [168, 169]. The release of siRNA could be monitored synchronously through the signal of energy resonance transfer. Xing et al. used UCNPs as energy transfer receptors through the FRET to control the release of luciferase. First, luciferase was encapsulated in a NIR-sensitive polymer, then through UCNPs to transfer the energy of luciferase to achieve the bioluminescence in the deep tissue of mice, which used for real-time imaging monitoring of tumor growth [144]. In the work of Liu et al., PEG-modified UCNPs were combined with terminal FA molecules and the chemotherapeutic drug DOX was incorporated into the surface of UCNPs through hydrophobic interaction to stimulate multifunctional targeting and cell imaging [32, 170]. For some intractable diseases, the combination of PDT and gene therapy is often chosen. For example, Zhao et al. developed a photodynamic therapy strategy for a strongly positively charged cationic conjugated polyelectrolyte (CPE) combined with UCNPs. CPE can be used as a photosensitizer for PDT and nucleic acid carrier to load siRNA (in Fig. 15), which gives the prepared UCNP@(CPEB) good stability and excellent siRNA loading capacity. They concluded that the efficient siRNA release (80%) and synergistic PDT treatment demonstrate suppressive effects on A549 tumors under 980-nm excitation [171].

#### Photodynamic therapy

**Fig. 16:**
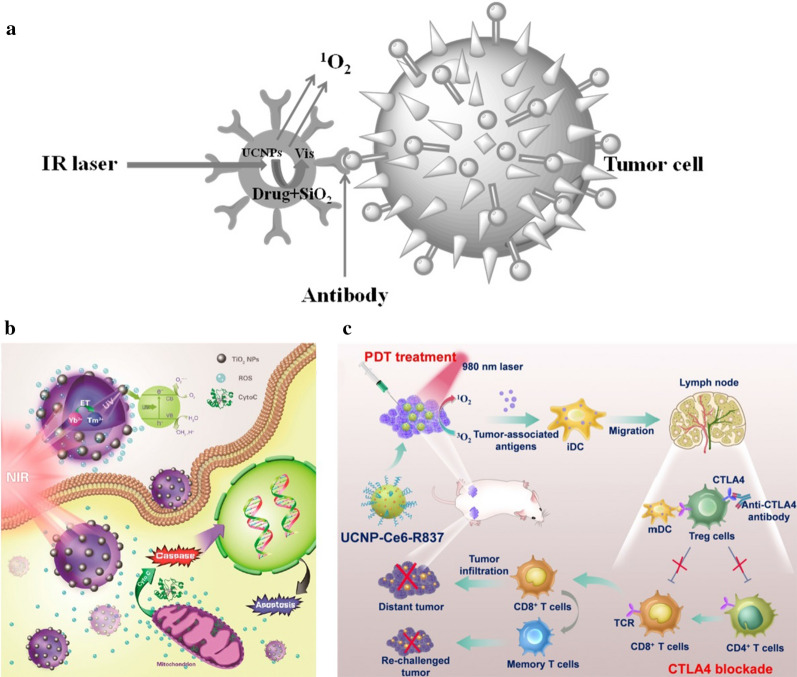
**a** Schematic illustration of the design of UCNP based drug delivery system for photodynamic therapy. **b** Plot for the potential molecular mechanism of inducing apoptosis with UCNPs@TiO_2_-based NIR light-mediated PDT treatment [176]. **c** Scheme summarizing the mechanisms of combining NIR-mediated PDT with CTLA-4 checkpoint blockade for cancer immunotherapy. UCNP-Ce6-R837 nanoparticles under NIR light would enable effective photodynamic destruction of tumors [116]. ( Copyright 2015, 2017. American Chemical Society. Reproduced with permission.)

PDT, an emerging technology for the diagnosis and treatment of diseases, uses photodynamic effects [172]. Briefly, the main treatment process is as follows: the photosensitizer is excited to generate active oxygen – for example, singlet oxygen (^1^O_2_) – entering the organism; thereafter, it can destroy tumor cells and achieve the purpose of cancer treatment [173]. Compared to UV and visible light, NIR light has a significant advantage in penetrating human tissues, making UCNP an ideal candidate for PDT applications in deep tissue cancer therapy. Zhang et al. encapsulated the photosensitizing molecule ZnPc in the lipid micelle-modified UCNP [174]. When irradiated with 980 nm excitation, ZnPc produced ^1^O_2_, which in turn killed cancer cells and detected tumor growth. Besides, the PDT which was guided by UCNPs synergistic with siRNA for tumor therapy made remarkable progress. Zhang et al. established a novel UCNP drug delivery system for the delivery of siRNA. They designed a delivery system that could be wrapped in light-tear polymer tape for efficient siRNA delivery under NIR modulation [175]. When the photocleavable linker (PhL) and PEG film are irradiated at 980 nm, the UCNP tear off the PEG film to stimulate the activation of HA to release reactive oxygen species (ROS) in order to assist siRNA in effectively increasing gene-silencing efficiency and inhibiting tumor cell growth in vivo and in vitro simultaneously [147]. As Fig. 16 illustrates, Hou et al. reported a novel NIR photosensitizer based on TiO_2_ coated UCNP core–shell nanocomposites (UCNPs@TiO_2_ NCs), which could be triggered with PDT and effectively inhibit tumor growth in tumor-bearing mice because it provided better tissue penetration than UV radiation [176]. Xu et al. used a UCNP loaded with chlorin e6 (Ce6) as a photosensitizer and imiquimod (R837) as a Toll-like receptor-7 agonist. The obtained multifunctional UCNP-Ce6-R837 enhanced tissue penetration depth under NIR irradiation and this not only achieved photodynamic destruction of tumors, but also generated tumor-associated antigen libraries under the condition of adjuvant, which further promotes a strong antitumor immune response [116].

#### Antibacterial photodynamic therapy on antibacterial and biofilms

**Fig. 17:**
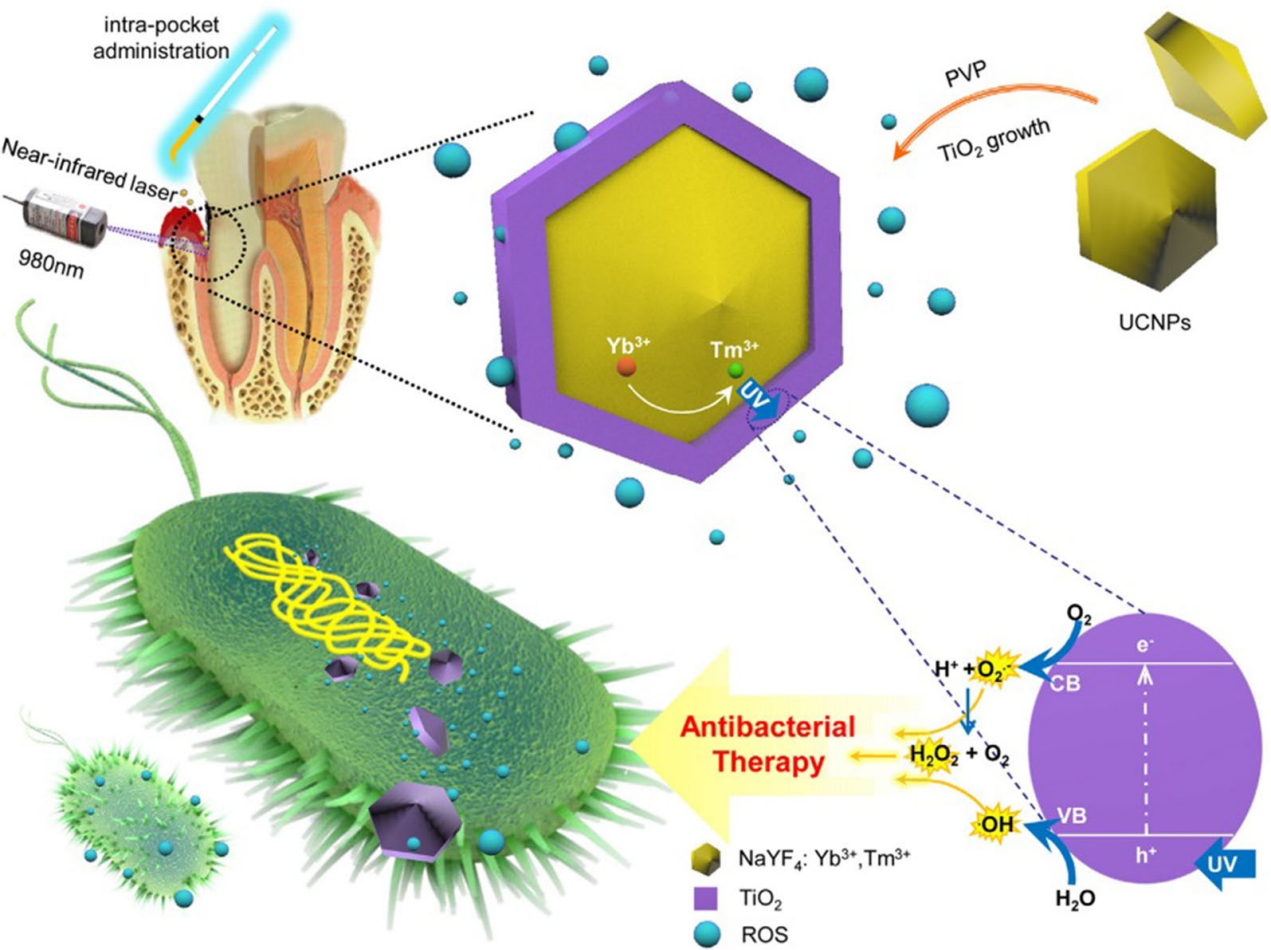
Synthesis of NaYF_4_:Yb^3+^,Tm^3+^@TiO_2_ and mechanism of aPDT under NIR irradiation. Upon NIR irradiation, the UCNP nucleus converts NIR light into ultraviolet (UV) light and the UV light in turn excites TiO_2_ to generate active oxygen, which eventually causes oxidative damage against the microorganisms. ( Copyright 2019. Elsevier. Reproduced with permission.)

At present, more than 80% of human infectious diseases are related to bacterial biofilms. Biofilms can effectively protect bacteria and produce antibiotic resistance. The emergence of super-resistant bacteria and the growth of bacterial biofilms have seriously hindered the development of biomaterials. Although inorganic materials such as Ag^+^ and Cu^+^ have excellent antibacterial activity, the expensive cost and the toxicity caused by excessive use is a crucial problem. It is necessary to carry out simultaneous biofilm removal and sterilization synergistic treatment. aPDT drug delivery systems based on UCNPs have gradually emerged in the filed. Yue and colleagues prepared UCNPs consisting of cationic N-octyl chitosan (OC)-coated UCNPs loaded with the photosensitizer zinc phthalocyanine (OCUCNP-ZnPc) from NaYF_4_, Yb, or Er core. They consist of a NaYF_4_ shell coated with chitosan and containing ZnPc, which exhibited excellent aPDT efficacy. Periodontitis is an infectious disease caused by bacterial biofilms that is usually treated by physically removing plaque with high recurrence. The aPDT method guided by UCNPs provides a novel solution to this situation. Qi and colleagues constructed an aPDT using NaYF_4_: Yb^3+^, Tm^3+^@TiO_2_ core–shell nanoparticles (Fig. 17). Under 980-nm excitation, UCNPs can emit strong UV light, which triggers the aPDT function of shell TiO_2_ through energy transfer, thereby achieving significant antibacterial effects on three periodontitis-related pathogens and biofilms [177]. In addition, Zhang et al. combined the photosensitizer Ce6 molecule with NaYF_4_: Yb, Er by hydrophobic interaction to overcome the problem of insufficient penetration depth of PDT treatment in deep periodontal tissues [178]. Subsequently, Mn was doped to enhance red fluorescence, and a significant inhibitory effect on biofilms of pathogenic bacteria such as *Porphyromonas gingivalis* and *Clostridium nucleatum* was observed under NIR excitation. At present, our group is constructing an aPDT platform based on UCNPs, which is related to esophageal diseases for the removal of *Porphyromonas gingivalis* and its derived biofilms, and further studies on the relationship between *Porphyromonas gingivalis* and esophageal diseases. Taken together, NIR-triggered UCNPs aPDT has demonstrated exciting potential in the treatment of a variety of oral infectious diseases, which has broad application prospects for other bacterial infection treatments.

## Conclusions and future perspectives

In this review, we briefly described the structure and energy level principle of UCNPs and introduced several methods for the synthesis and modification of UCNPs based on chemical matrix reactions. Lanthanide-based UCNPs play an excellent role in biomedical applications, especially bioimaging and disease treatment, due to their unique properties. Concerning drug delivery and release, UCNPs outperformed effectively PDT for tumors and exhibited promising clinical value. At the same time, the use of NIR light in UCNP-based aPDT in antibacterial and biofilm removal applications was a major innovation for infectious disease treatment. On the other hand, the practical applications of UCNPs still face formidable challenges that will require researchers in all disciplines to work together closely. Although these luminescent UCNPs have increased the sensitivity of biosensing and bioimaging and have photoinitiated biological and chemical reactions, they still have limited upconversion efficiencies, and considerable effort has been devoted to upconversion efficiency improvement. The narrow-band NIR absorption can be expanded to a more suitable wavelength range using dye-sensitized UCNPs; however, it was extremely unstable both in vivo and in vitro. Nevertheless, the biomedical applications of UCNPs have been expanded in recent years despite the enormous challenges in practice. We still have reason to believe that multifunctional lanthanide-based upconversion luminescent nanomaterials have a bright future in biomedical and related fields.

## Data Availability

Please contact author for data requests.
